# Diverse Genome Structures among Eukaryotes May Have Arisen in Response to Genetic Conflict

**DOI:** 10.1093/gbe/evae239

**Published:** 2024-11-07

**Authors:** Elinor G Sterner, Auden Cote-L’Heureux, Xyrus X Maurer-Alcalá, Laura A Katz

**Affiliations:** Department of Biological Sciences, Smith College, Northampton, MA 01063, USA; Department of Biological Sciences, Smith College, Northampton, MA 01063, USA; American Museum of Natural History, Department of Invertebrate Zoology, Institute for Comparative Genomics, New York, NY, USA; Department of Biological Sciences, Smith College, Northampton, MA 01063, USA; Program in Organismic and Evolutionary Biology, University of Massachusetts Amherst, Amherst, MA 01003, USA

**Keywords:** eukaryogenesis, genome dynamics, polyploidy, aneuploidy, genetic conflict, meiosis

## Abstract

In contrast to the typified view of genome cycling only between haploidy and diploidy, there is evidence from across the tree of life of genome dynamics that alter both copy number (i.e. ploidy) and chromosome complements. Here, we highlight examples of such processes, including endoreplication, aneuploidy, inheritance of extrachromosomal DNA, and chromatin extrusion. Synthesizing data on eukaryotic genome dynamics in diverse extant lineages suggests the possibility that such processes were present before the last eukaryotic common ancestor. While present in some prokaryotes, these features appear exaggerated in eukaryotes where they are regulated by eukaryote-specific innovations including the nucleus, complex cytoskeleton, and synaptonemal complex. Based on these observations, we propose a model by which genome conflict drove the transformation of genomes during eukaryogenesis: from the origin of eukaryotes (i.e. first eukaryotic common ancestor) through the evolution of last eukaryotic common ancestor.

**Significance**

The focus on limited “model” lineages of animals, plants, and fungi has led to the idea of a typified eukaryotic life cycle that alternates between haploid and diploid stages. These “textbook” depictions present tidy models that focus on the dominance of diploid phases in animals and haploid phases in fungi so that even the alternation of generations in plants (i.e. the presence of mitotic divisions in both haploid and diploid phases) is often presented as an exception. Moreover, these simplistic haplo-diploid life cycles underlie most population genetic models that estimate the effect of evolutionary forces (i.e. selection and drift). Yet, the expansion of genome-scale sequencing efforts, coupled with the rich but often overlooked literature on diverse microeukaryotic lineages that emerged from microscopy studies, reveals a tremendous diversity of eukaryotic life cycles that extend beyond simple haploid-diploid transitions and necessitates revising models on the origin of eukaryotic life.

## Introduction

**Fig. 1.**
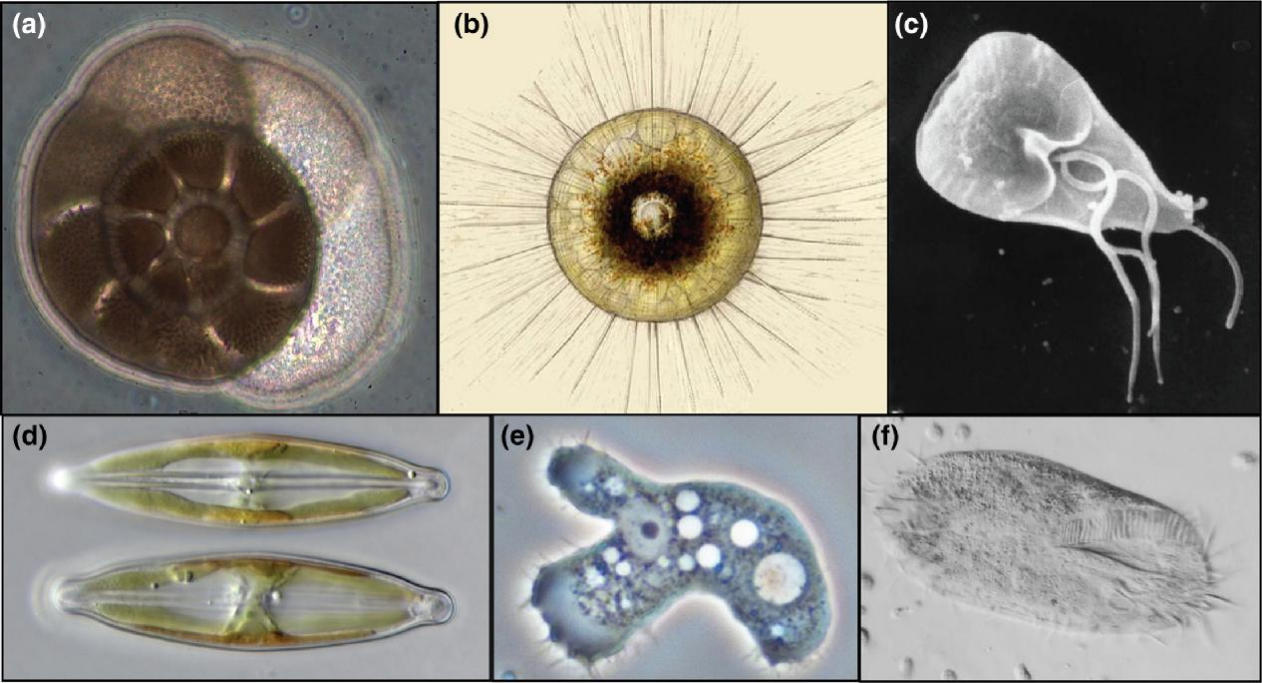
Examples of diverse microbial eukaryotes discussed in parts I and II. a) *Ammonia* (Rhizaria, Foraminifera), isolated in our lab; b) *Aulacantha* (Rhizaria, formerly radiolaria); c) *Giardia* (“Excavata”); d) Naviculoid diatom (Stramenopila); e) *Acanthamoeba* (Amoebozoa); f) *Oxytricha* (Alveolata, Ciliophora). Images b)–f) accessed from the internet and all available under CC3 license.

**Fig. 2.**
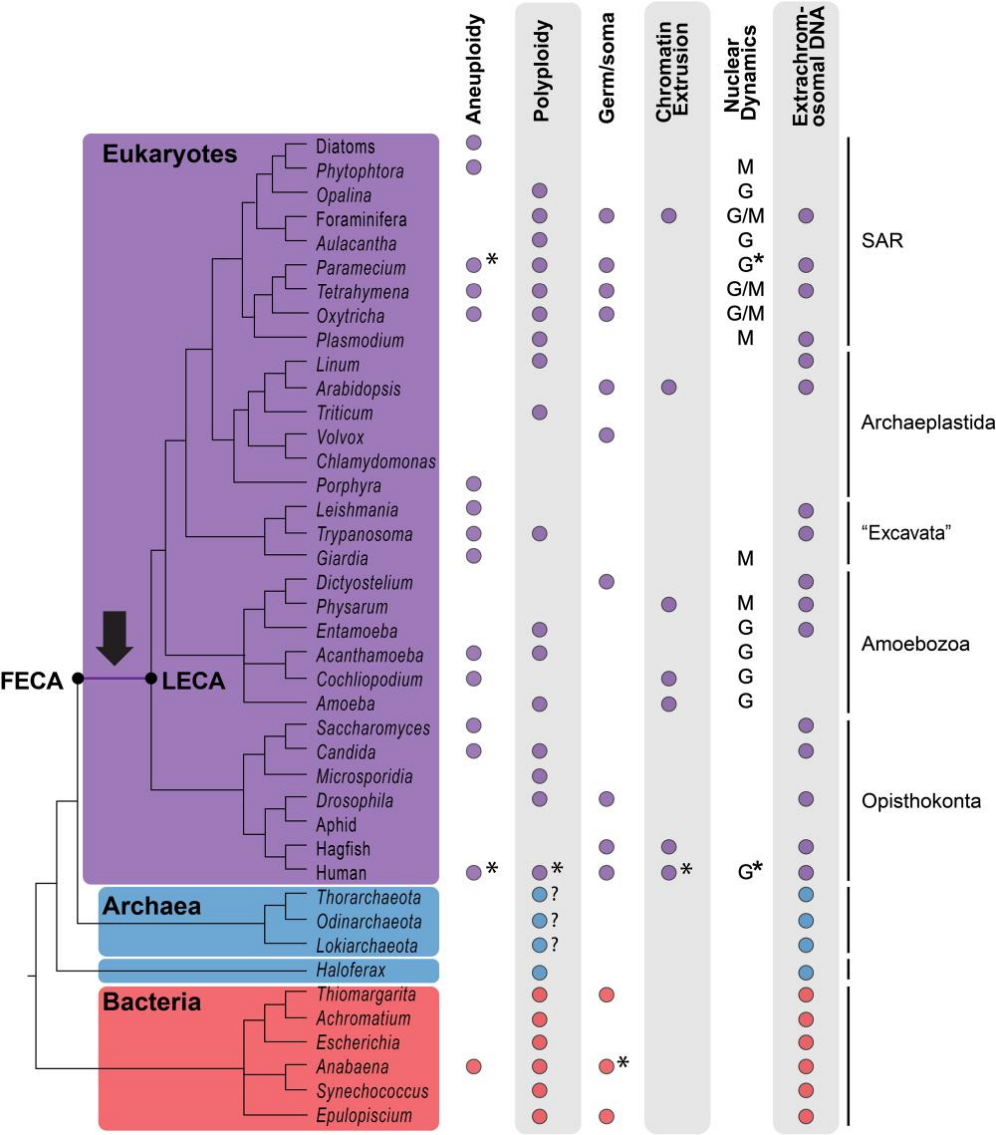
Taxonomy of organisms discussed with eukaryotes (purple) shown as nested among the archaea (blue) and sister to bacteria (red). We document genomic features associated with instability that are present throughout the tree of life, particularly among eukaryotes (see part I). The tree is rooted on opisthokonts as suggested by Cerón-Romero et al. (2022), though this is controversial; an alternative hypothesis is that the root lies among the “Excavata” (taxon in quotes as monophyly unclear; Al Jewari and Baldauf 2023). Absences of a feature may represent a lack of data (common among microeukaryotes) rather than true absences. Colored boxes on the tree encircle monophyletic clades. G, growth; M, multinucleate; *, somatic. As discussed in the text, the question mark indicates the inferences of polyploidy in Asgard (and other) archaea. Arrow points to the FECA to LECA transition (i.e. eukaryogenesis) as discussed in the text.

The bulk of eukaryotic diversity is microbial, with plants, animals, and fungi representing just 3 multicellular lineages that fall among a plethora of diverse clades of microeukaryotes (e.g. Fig. 1; Adl et al. 2019). With eukaryotes nested within Asgard archaea (Williams et al. 2020; Eme et al. 2023), they are perhaps best considered as a lineage of Archaea that acquired mitochondria and subsequently evolved features such as the nucleus and complex cytoskeleton (Roger et al. 2021; Donoghue et al. 2023; Eme et al. 2023). Major eukaryotic lineages include Opisthokonta (including animals and fungi) and Archaeplastida (including plants) plus several other major clades (e.g. Alveolata, Amoebozoa, Stramenopila, and Rhizaria) that are predominantly microbial (Adl et al. 2019; Burki et al. 2020; Fig. 2). Many biological principles originate from studies of just a few macrobial lineages and neglect data from microeukaryotes. Among microeukaryotes, the bulk of life history studies have come from pathogens (e.g. *Entamoeba*, *Plasmodium*, and *Phytophthora*) and a few free-living model lineages such as *Tetrahymena* and *Chlamydomonas* (Chalker 2008; Jinkerson and Jonikas 2015). Here, we present insights on genome properties from diverse extant non-model lineages of microeukaryotes and discuss their impact on our understanding of early eukaryotic evolution.

We divide this perspective into 3 sections: (i) a survey of genome dynamics and noncanonical life cycles; (ii) a brief review of eukaryotic innovations that underlie these dynamics; and (iii) a presentation of a model by which genetic conflict drove eukaryogenesis and enabled the genome dynamics observed among extant eukaryotes. Genetic conflict refers to instances where genes, or genetic elements more broadly, compete within the same nucleus (or between nuclei within a cell) for transmission into the next generation, with examples including transposable elements and meiotic drive (Burt and Trivers 2008). We argue that the diverse instances of unstable genomic systems surveyed in part I and now regulated by the innovations described in part II are evidence of an underlying driver of evolution that became exaggerated in eukaryotes prior to the last eukaryotic common ancestor (LECA), namely, genetic conflict. In other words, we posit that the merger of bacterial and archaeal genomes exacerbated the existing conflict (i.e. between host and mobile genetic elements [MGEs]) to drive genome evolution in eukaryotes.

We discuss eukaryotic genome dynamics as examples of “somatic” functions in lineages that maintain vertical inheritance of germline material despite highly flexible genome features. The distinction between germline and somatic genomes is clearest in animals that sequester germline cells (Extavour and Wilkins 2008) and in 2 microeukaryotic lineages—ciliates and some Foraminifera—that can have distinct somatic and germline nuclei within the same cell (Goetz et al. 2022; Maurer-Alcala and Nowacki 2019; Rzeszutek et al. 2020; Fig. 2). Here, we expand on the idea that germline-soma distinctions also occur within nuclei, allowing a portion of the genome (i.e. the soma) to be dynamic while germline material is marked by epigenetic mechanisms for inheritance (e.g. Parfrey et al. 2008; Collens and Katz 2021).

## Part I: The Dynamic Eukaryotic Genome

We describe examples of noncanonical genome features exemplified across diverse eukaryotic lineages, focusing on genome endoreplication, aneuploidy, chromatin extrusion, and maintenance of extrachromosomal DNA (Table 1 and Fig. 2). The existence of such genome dynamics is consistent with the possibility that LECA had a dynamic genome in which epigenetic marks distinguished germline and somatic material, which we and others have argued shaped the early eukaryotic genomes (Aravind et al. 2012; Koonin 2017; Collens and Katz 2021). While the specifics of the dynamic processes discussed below may reflect convergent evolution, together they suggest that germline-soma distinctions represent the resolution of genetic conflict in early eukaryotic evolution, with the former a mechanism to both protect against and harness the power of the genetic conflict.

**Table 1:** . Glossary of terms

Amitosis	Literally, not mitosis; a stochastic division of chromatin/chromosomes in the absence of mitotic spindles and centromeres. Can result in haphazard division of genetic material
Asgard clade	A clade of archaea whose members share a set of genes that were previously believed to be unique among eukaryotes; eukaryotes appear to be a lineage within the Asgard archaea
Microeukaryotes (or protists)	All eukaryotes that are microscopic, most are unicellular (e.g. amoebae, flagellates, and ciliates), and they do not form a single evolutionary group as plants, animals, and fungi nest among microbial lineages
Eukaryogenesis	The evolution that occurred starting at the merger event between a bacterium and archaeon (producing FECA) and ending at LECA
Endoreplication	Duplication of the whole or parts of the genome without division of the nucleus
Aneuploidy	Unequal replication of different regions of the genome. This can range from different numbers of whole chromosomes to small segments
Extrachromosomal DNA	DNA stored outside of chromosomes, including plasmids
Chromatin extrusion	Removal, “cleansing,” or other forms of loss of chromatin from the nucleus
Chromatin diminution	Similar to chromatin extrusion, a process identified in the literature as a diminution of genome content
Genetic conflict	The result of 2 genetic/genomic components whose inheritance is intertwined such that increased frequency of one can lead to the decrease of the other
Micronuclei	Small nuclear structures that occur in addition to the primary nucleus and that can contain portions of the genome. Differs from the germline nucleus of ciliates, also known as a micronucleus
Monoploidy	The presence of a single chromosome copy
Autogenous origin of the nucleus	The theory that the nucleus arose from within eukaryotes, rather than through symbiosis
Segregation load	Effect making polypoid (or diploid) individuals more fit because less beneficial alleles are over dominated by the more beneficial one

### Endoreplication and Ploidy Cycles

**Fig. 3.**
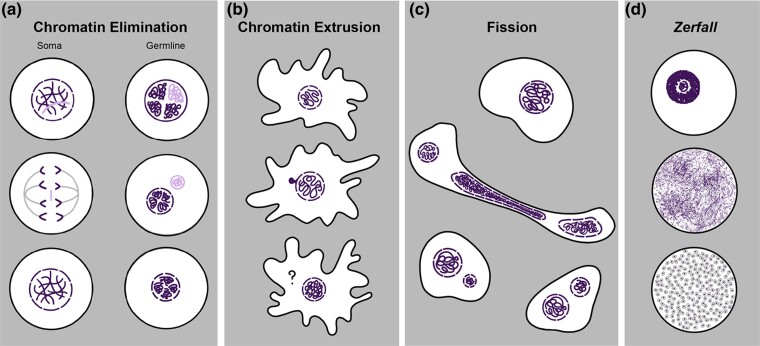
Examples of noncanonical chromatin dynamics in diverse lineages, which suggest a division between germline and somatic material within nuclei. a) Chromatin elimination from soma in the form of B chromosomes and from the germline as occurs during paternal germline elimination in some insects (see Dedukh and Krasikova 2022). b) Extrusion of compact chromatin in *A. proteus* based on images from Goodkov et al. (2020). c) Fission of genetic material in *Cochliopodium*, with purple representing the DAPI stained nucleus from Tekle et al. (2014). d) *Zerfall*, in *Allogromia laticollaris* (Foraminifera); here, DNA at an estimated 11,000 N is extruded throughout the cytoplasm prior to the formation of gametic nuclei; illustration based on Timmons et al. (2024). Artwork by Tejasvi Kumaran (Smith College).

Haplo-diploidy is not the rule for many species, and common mechanisms of polyploidization in eukaryotes include hybridization, whole genome duplication, and endoreplication (i.e. amplification of genome content). While the first 2 of these processes are relatively well described (e.g. Gerstein and Otto 2009; Van de Peer et al. 2017), the mechanisms and evolution underlying endoreplication remain understudied, despite its prevalence in diverse lineages (Fig. 2). Ploidy levels that show large cyclical variation throughout the life cycle have been documented in the amoebozoan lineages *Entamoeba* (Mukherjee et al. 2009) and *Amoeba proteus* (Berdieva et al. 2019). In *Entamoeba*, DNA content varies substantially with as much as a 40-fold increase across life history stages (Mukherjee et al. 2008). In *A. proteus*, polyploidization through endoreplication (5- to 6-fold) is followed by chromatin extrusion, a process by which DNA is expelled from the nucleus out into the cytoplasm (Demin et al. 2020; Goodkov et al. 2020; Fig. 3b).

Polyploidy is well documented in the somatic macronuclei of ciliates (Alveolata) in which chromosome copy number can reach >1,000 N (Wancura et al. 2018; Maurer-Alcala and Nowacki 2019) and in Foraminifera (Rhizaria) where genome content increases through endoreplication between life history stages, including haploid and diploid phases (Timmons et al. 2024). *Aulacantha scolymantha* (Rhizaria) reaches ∼2,000 N through endoreplication and then reduces its genome upon division by sporulation (Grell 1953; Lecher 1978). Endoreplication also occurs in somatic cells of plants and animals, leaving germline material untouched while generating large cells with high genome copy number in various organs (Orr-Weaver 2015; Shu et al. 2018).

Assessment of genome content indicates that polyploidy is both common and potentially beneficial in diverse bacteria and archaea (Santer et al. 2022; Soppa 2022; Ionescu et al. 2023; Özer et al. 2024), indicating some level of genome flexibility that predates eukaryogenesis. Polyploidy underlies the increase in cell size in the cyanobacterium *Synechococcus elongatus* and other diverse gigantic bacteria (Angert 2021; Ionescu et al. 2023). Polyploidy is common in halophilic archaea and may promote survival in extreme conditions (e.g. intense UV and/or high salinity; Jaakkola et al. 2014). Even *Escherichia coli* remains monoploid only under slow growth conditions and becomes oligoploid under optimal growth (Pecoraro et al. 2011).

### Aneuploidy

Aneuploidy refers to differences in copy number between chromosomes and chromosomal regions within a single nucleus (Torres et al. 2008). It is most widely understood in the context of animal diseases (e.g. in cancer cells; Salmina et al. 2019), leading to the view that aneuploidy is primarily deleterious (Torres et al. 2008). However, aneuploidy occurs within the life cycles of a variety of eukaryotic lineages and can generate genetic variability (Sterkers et al. 2014). In the plasmodial life stage of the slime mold *Physarum*, diploid nuclei produce viable spores (i.e. germline cells), while haploid nuclei in amoeboid phases generate aneuploid nuclei by “pseudomeiosis” (Holt 1980; Turner and Goodman 1981). Similarly, mosaic aneuploidy, when ploidy of chromosomes or chromosomal regions varies within a population, is common in the parasite *Leishmania* (Negreira et al. 2022) and in the water mold *Phytophthora*; different isolates exhibit different allele frequencies across loci (Hu et al. 2020). More broadly, aneuploidy may be adaptive, providing variability, allowing “complementation” between cells, and enabling amplification of beneficial mutations (Sterkers et al. 2012, 2014).

Aneuploidy is perhaps most extreme in the binucleate diplomonad genus *Giardia* (Le Blancq and Adam 1998; Carpenter et al. 2012; Tůmová et al. 2019). Here, karyotypes vary in terms of both chromosome number and length, with complex patterns between strains and species as well as within populations (Le Blancq and Adam 1998; Tůmová et al. 2016). Le Blancq and Adam (1998) observed homologous chromosomes of varied lengths in a single strain, and more recently, Tůmová et al. (2016) used fluorescence *in-situ* hybridization to identify uneven chromatid segregation resulting in aneuploidy. *Giardia*'s karyotype instability is facilitated by extensive subtelomeric rearrangements, which may permit rapid host colonization (Tůmová et al. 2016)*. Giardia* appears to have lost canonical meiosis, depending instead on a novel mechanism of chromosome regulation during cyst stages (Carpenter et al. 2012).

### Extrachromosomal DNA

The classic “textbook” depiction of eukaryotic DNA is a set of large linear chromosomes capped with telomeres and marked by centromeres. However, eukaryotic genomic material is organized in a plurality of states, from short extrachromosomal circular DNA (eccDNA) to supernumerary material such as B chromosomes and plasmids (Burt and Trivers 2008; Zuo et al. 2022). First discovered in 1964, eccDNA varies in size from 0.15 to 100 kb (Wang et al. 2021) and is generated through a variety of mechanisms including chromothripsis (i.e. shattering and extensively rearranging chromosomes) and circularization of excised chromosomal regions (Ling et al. 2021; Zuo et al. 2022). Extrachromosomal rDNA is found widespread among eukaryotes (Torres-Machorro et al. 2010; Cao et al. 2021; Ling et al. 2021), and some lineages including *Naegleria* (Nguyen et al. 2021) and *Entamoeba* (Sehgal et al. 1994; Bhattacharya et al. 2000) appear to lack *chromosomal* rDNA altogether (reviewed in Torres-Machorro et al. (2010)).

The available data suggest that some eccDNA may have regulatory roles, for example, increasing expression of a gene by increasing its copy number, whereas others may drive genomic rearrangements through reinsertion at varying sites (Pavri 2017; Turner et al. 2017). Interestingly, eccDNA may also play a role in stabilizing chromosome structures as it can be composed of centromeric elements and can aid in lengthening telomeres, as observed in somatic animal cells (Neumann et al. 2013). In contrast, the 2 µm plasmid of yeast has no known function other than ensuring its own inheritance (Burt and Trivers 2008; McQuaid et al. 2019). Similarly, supernumerary B chromosomes are not necessary for organismal survival but are nonetheless preferentially inherited, a phenomenon termed “chromosome drive” that is an example of genetic conflict (Burt and Trivers 2008).

### Chromatin Extrusion and Other Noncanonical Chromatin Dynamics

Chromatin extrusion and similar processes that eliminate chromatin from a nucleus occur in diverse eukaryotic lineages as an integral part of their life cycles. Chromatin extrusion has been documented in the genus *Amoeba* (Berdieva et al. 2019; Goodkov et al. 2020), and fluorescence microscopy in *Cochliopodium* demonstrates the fission of polyploid nuclei inherent within the life cycle (Tekle et al. 2014; Fig. 3c). In Foraminifera, a process termed *Zerfall* refers to the “decay” of a large endoreplicated haploid nucleus, during which genetic material is distributed throughout the cytoplasm prior to gametogenesis (Goldstein 1997; Timmons et al. 2024; Fig. 3d). Chromatin is also removed during the development of somatic macronuclei in ciliates (Noto and Mochizuki 2017; Rzeszutek et al. 2020) and in the establishment of somatic nuclei of diverse animals (e.g. copepods, hagfish, nematodes; Suh and Dion-Côté 2021; Dedukh and Krasikova 2022).

DNA elimination also occurs in situations of stress and disease (Dalla Benetta et al. 2020; Shapiro 2021; Dahiya et al. 2022). In plants, DNA elimination often follows hybridization where it is associated with the presence of divergent centromeric histones (Tan et al. 2015; Comai and Tan 2019). DNA is also eliminated in response to stress in some varieties of flax, with an estimated 15% of the genome (e.g. repetitive elements) removed within a single generation (Cullis and Cullis 2019). DNA deletion also occurs in prokaryotes in response to stress and is often mediated by mobile genetic elements (Cao et al. 2022; Paul and Eren 2022).

Originally described in *Parascaris* in 1887, the generation of somatic nuclei can include large-scale genome rearrangements that include the elimination of substantial regions of the germline genome (Boveri 1887; Wang and Davis 2014). In ciliates, the diploid germline genome remains predominantly quiescent in its own “micronucleus” while polyploid somatic genomes are generated through complex epigenetically regulated processes following conjugation (reviewed in Maurer-Alcala and Nowacki (2019) and Rzeszutek et al. (2020)). This phenomenon is particularly striking in the ciliate *Oxytricha trifallax*, where >90% of the germline genome is eliminated during development of a new somatic genome; here, processing includes stitching together >220,000 DNA segments into its ∼16,000 unique somatic chromosomes (Chen et al. 2014). Through analyses of transcriptome data from diverse lineages, we have demonstrated that patterns of molecular evolution in ciliates correspond to genome architecture as we find more diverse gene families in lineages with extensively processed somatic genomes (Maurer-Alcalá et al. 2018, 2024; Yan et al. 2019). We argue that the combination of endoreplicated somatic chromosomes and amitosis (i.e. division without strict regulation of chromosome complements; Table 1) along with the breakdown of linkage groups in ciliate macronuclei underlies the unusual patterns of molecular evolution (Zufall et al. 2006; Maurer-Alcalá et al. 2018).

## Part II: Enigmatic Origins of Eukaryotic Features

Here, we explore the origins of eukaryotic features that underpin the dynamic processes described in part I, focusing on the enigmatic origins of the nucleus, centromeres, and the synaptonemal complex, the latter responsible for pairing homologous chromosomes in meiosis. Together, these features provide the basis for dynamic genome regulation, with the latter 2 playing critical roles in the inheritance of full genome complements (Wickstead and Gull 2011; Gabaldon 2021; Park and Leroux 2022). Our focus is only a subset of eukaryotic features and particularly those that potentially contribute to genome conflict (e.g. centromeres competing for spindle fibers and synaptonemal complex regulating karyotypes) as discussed in part III. We direct readers to other papers that cover further examples, including hypotheses as to the genomic content of the first eukaryotic common ancestor (FECA) and LECA (e.g. Pittis and Gabaldón 2016; Roger et al. 2021; Vosseberg et al. 2021; Donoghue et al. 2023).

Despite its importance, the dynamics around the origin of the nucleus—the defining feature of eukaryotes—remain unclear. Numerous hypotheses have been proposed for an autogenous origin of the nucleus that focus on the origin of membranes and the putative homologs in archaeal lineages (reviewed in Baum and Spang (2023) and Donoghue et al. (2023)). Others have pointed to similar structures in a few bacteria (Boedeker et al. 2017; Volland et al. 2022) and even viruses (Takemura 2020) in which DNA is associated with internal membranes, though these are perhaps more likely the result of convergence given the phylogenetic placement of these lineages. A key feature of the nuclear membrane is that it separates transcriptional machinery from translational machinery, suggesting the possibility that the invasion of introns or other mobile elements in early eukaryotic evolution contributed to the origins of this structure (Martin and Koonin 2006; Martin et al. 2015).

Centromeres have a critical role in the management of chromosomes, including the faithful segregation of chromosomes during nuclear division. Centromeres are argued to have evolved from telomeres, which in turn evolved from “islands of transposable elements,” signature agents of genome conflict (Villasante et al. 2007; Kumon and Lampson 2022). Indeed, hypotheses in the literature propose that genome conflict drove the evolution of centromeres, whereby transposable elements (TEs) and other selfish elements compete to “cheat” as a means of increasing their inheritance by taking advantage of regions without recombination while the host cell attempts to regulate these features (Kumon and Lampson 2022). Recent work in *Drosophila* has tied TE activity to centromere formation and maintenance, representing a genomic “mutualism” where TEs escape extinction by conferring stable centromeric regions for faithful chromosome segregation (Hemmer et al. 2023). TE-enriched centromere structures are common across eukaryotes, as exemplified in the amoebozoan *Dictyostelium discoideum*, where TEs comprise ∼86% of the centromeres (Glöckner and Heidel 2009). The biased insertion of TEs near centromeres is believed to drive kinetochores’ rapid evolution, where only a subset of proteins have recognizable prokaryotic homologs (e.g. Tromer et al. 2019).

The synaptonemal complex, a rapidly evolving yet fundamental eukaryotic feature (Hemmer and Blumenstiel 2016), is hypothesized to be a key outcome of genomic conflict (e.g. centromeric and meiotic drive) during the evolution of machinery for chromosome segregation. The synaptonemal complex, which underlies the formation of tetrads during meiosis, is an indicator of eukaryotic sex (i.e. meiosis and syngamy) and is also believed to play a role in the evolution of ploidy control (Maciver 2019). While some components of the meiotic toolkit are highly conserved among extant taxa, the protein complexes comprising the synaptonemal complex evolve much more rapidly (Maciver et al. 2019; Grishaeva and Bogdanov 2021). This can be extreme, as in the Symbiodiniaceae dinoflagellates, where commonly conserved synaptonemal complex protein-coding genes (e.g. “Zip” genes) are reported missing, whereas most other meiosis-related genes are present (Shah et al. 2020). In other lineages, components of the synaptonemal complex have been repurposed, such as in kinetoplastids where they are components of kinetochores (Tromer et al. 2021). Additionally, in the hyper-polyploid (∼2,000 N) rhizarian *Aulacantha*, the synaptonemal complex plays fundamental roles in depolyploidization and generation of putatively haploid (N) daughter cells (Lecher 1978).

## Part III: Genome Evolution During Eukaryogenesis—The FECA to LECA Transition

Here, we present a model for genome evolution through the lens of inter- and intragenomic conflict, which we and others have argued shaped early eukaryotic genomes (Aravind et al. 2012; Koonin 2017; Collens and Katz 2021) and which we posit drove the evolution of mechanisms to distinguish between an epigenetically marked germline and more flexible somatic genetic material. Reconstructing events at the origin of eukaryotes is challenging given the tremendous amount of time that has elapsed, extinction events erasing early innovations, and subsequent descent with modification that generated extant biodiversity. Despite these challenges, inferences can be made as to the nature of LECA and, into even deeper time, FECA. We argue that genome conflict was exaggerated when a bacterium and an archaeon merged at the beginning of eukaryogenesis, leading to the evolution of eukaryotic chromosomes with telomeres that stabilize chromosome structure, centromeres that compete for spindles during nuclear division, and ultimately karyotypes that are organized through meiosis and that serve as the basis for defining eukaryotic species.

**Fig. 4.**
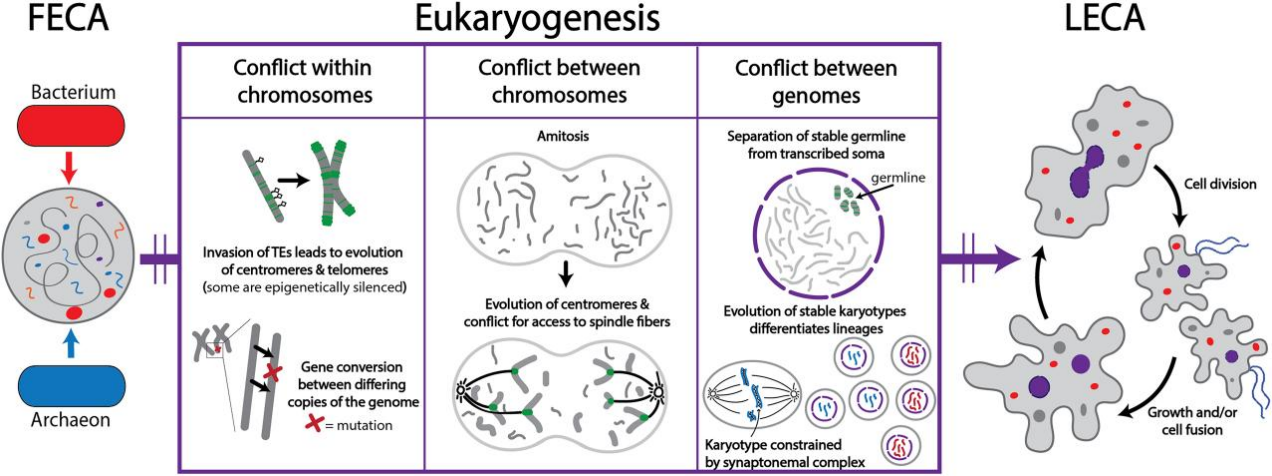
Our hypothesis on the role of genetic conflict during eukaryogenesis (i.e. the FECA to LECA transition), including in driving genome structures and ultimately the evolution of karyotypes. We propose that eukaryotic innovations arose as a response to the conflict between genomes plus an influx of MGEs (e.g. transposable elements [green] and viruses) at the fusion of a bacterium (orange) and an archaeon (blue), which led to both intra- and intergenomic conflict. Conflict within chromosomes led to the evolution of both telomeres and centromeres (see text part I; left panel) as well as an expansion of mechanisms for gene conversion that can remove deleterious alleles. We speculate that early eukaryotes divided genetic material randomly through amitosis (see Table 1) and that subsequent competition among chromosomes resulted in the evolution of centromeres that compete for spindle fibers (middle panel). At some point, eukaryotes evolved nuclei (purple dashed circle) along with mechanisms to distinguish germline (inherited) from somatic (expressed) genetic material, and that the concomitant evolution of the synaptonemal complex enabled the establishment of karyotypes and subsequently the evolution of biological species (i.e. those that are reproductively isolated; right panel). These processes led to a complex LECA (far right image), likely an amoeboflagellate that could grow (by either increasing nuclear size and/or becoming multinucleated), fuse, and divide. Under such a scenario, the dynamic features observed among extant eukaryotes (Figs. 2 and 3) likely build from a combination of ancestral and recently evolved features that regulate distinctions between germline (i.e. carrying epigenetic marks) and somatic (i.e. polyploid, aneuploid, extrachromosomal/extruded DNA) genetic material.

Though the nature of the first eukaryote (FECA) is debated, phylogenomic analyses robustly place eukaryotes as a lineage of Archaea and “eukaryotic signature proteins” have been identified within the Asgard archaea (reviewed in Williams et al. (2020) and Eme et al. (2023)). Given this, we define eukaryogenesis as the period of evolution starting at the origin of the lineage (i.e. FECA) that emerged when an archaeal lineage acquired the bacterial symbiont that eventually gave rise to mitochondria, and ending in LECA, though we acknowledge the debates on the timing of mitochondrial acquisition (e.g. Pittis and Gabaldón 2016; Roger et al. 2021; Donoghue et al. 2023). The merger of the archaeal and bacterial genomes in FECA likely led to an expansion of mobile genetic elements (e.g. viruses and transposable elements), leading to corresponding elaboration of the epigenetic machinery present within the ancestral lineages (reviewed in Collens and Katz (2021); Fig. 4). Much of the resulting eukaryotic epigenetic toolkit, including modifications of histones and deployment of small RNAs as regulatory elements, appears to have been well diversified by LECA (Weiner et al. 2020).

We and others speculate that genomes in early eukaryotes were polyploid (or at least not in strict haploid-diploid cycles) given the prevalence of polyploidy in bacteria, archaea, and eukaryotes (see part I above; Fig. 4; Maciver 2016; Markov and Kaznacheev 2016; Santer et al. 2022; Soppa 2022; Van de Peer et al. 2017). Polyploidy can enable exploration of new niches due to greater metabolic flexibility associated with increased heterozygosity, and/or an increase in cell/body size associated with larger genomes (Melaragno et al. 1993; Edgar and Orr-Weaver 2001; Gerstein and Otto 2009). More broadly, cyclical polyploidy, which occurs among diverse extant microeukaryotes (see part I), may be beneficial as an increase in the frequency of ploidy cycles theoretically correlates with a reduction in mutation load (Kondrashov 1994). At the same time, polyploidy can mask deleterious mutations, leading to an increase in the frequency of deleterious alleles in a process termed an “evolutionary trap” (Gerstein and Otto 2009; Markov and Kaznacheev 2016). This effect can be mitigated if deleterious alleles are eradicated or beneficial alleles amplified under processes like aneuploidy (i.e. differential amplification of chromosome regions, chromatin extrusion, and DNA elimination) or through homologous recombination (e.g. gene conversion; Maciver 2019). Under this model, that machinery used for this homologous recombination may have been a precursor to machinery later employed in meiosis (Maciver 2019).

Polyploidy may have contributed to the genomic conflict that spurred the evolution of key eukaryotic features as different chromosome copies “complete” for representation in future generations. Maciver (2019) argues that meiotic machinery may be derived from a set of genes that prokaryotes evolved to regulate ploidy and facilitate homologous recombination, consistent with the observation of these processes in diverse archaea and bacteria (reviewed in Oliverio and Katz (2014) and Özer et al. (2024)). During eukaryogenesis (i.e. the FECA to LECA transition), and before the evolution of meiosis and mitosis, amitosis would have generated daughter cells with a diversity of genome contents that contributed to chromosome-level competition for inheritance. This, combined with the possibility that no daughter cells receive a full chromosomal complement in amitosis, may have driven the evolution of centromeres and related meiotic and mitotic machinery involved in regulating chromosome segregation in extant eukaryotes (Fig. 4). A further factor could be that amitotic division of polyploid cells led to potential costs *via* the breakdown of coadapted gene complexes during segregation/division (i.e. segregation load; Markov and Kaznacheev 2016). Multinuclearity, which has been inferred to be present in LECA (Skejo et al. 2021), could provide an additional buffer to the effects of polyploidization, with varying ploidy levels between nuclei creating a cell-wide balance.

As previously argued (Zufall et al. 2006; Parfrey et al. 2008; Weiner et al. 2020; Collens and Katz 2021), LECA may have relied on epigenetic mechanisms to mark a full genome complement as its germline, protecting this complement for inheritance while other portions of the genome remained dynamic (i.e. experiencing cyclical polyploidization, aneuploidy, chromatin extrusion; see part I). Marking a stable germline complement for inheritance would enable more efficient selection for coadapted gene complexes and mitigate the effects of genomic conflict. Importantly, intranuclear germline-soma distinction may have evolved as a mechanism not only to protect the vertically heritable material from genomic conflict but also in order to take advantage of these dynamics. For example, Zufall et al. (2006) argue that the distinction of germline and somatic nuclei in ciliates allows for differential expansion of beneficial alleles in the soma, and ultimately the accumulation of compensatory mutations in the germline, analogous to some population-level phenomena. Also, the evolution of the synaptonemal complex, which physically connects highly similar chromosomes, would further drive the evolution of stable karyotypes. Together, these phenomena—marking of a stable germline and the evolution of meiosis—contributed to the beginning of speciation within eukaryotes based on genetic exchange through sex (i.e. meiosis and karyogamy; Fig. 4). As a result, “biological” or “phylogenetic” species of eukaryotes emerged following the transition from FECA to LECA.

### Synthesis

Our synthesis of the data on genome dynamics from lineages sampled across the tree of life demonstrates tremendous flexibility that challenges “textbook” views of eukaryotic genomes as stable systems. The highly flexible life cycles among eukaryotes can include endoreplication of genomes, amplification of specific genomic loci, and elimination of others, processes that we argue led to the ability to distinguish germline from somatic genetic material even within a single nucleus. We hypothesize that genome dynamics in extant lineages, whether homologous or convergent, resulted from extensive genomic conflict during eukaryogenesis (i.e. the period of evolution between FECA and LECA), which elaborated on processes and mechanisms present among prokaryotic ancestors. By the origin of LECA, this conflict had selected for stable karyotypes (i.e. conserved chromosome numbers that are tightly regulated by the cytoskeleton and the synaptonemal complex) that now define biological species as actually or potentially interbreeding lineages. We predict that as researchers turn both the microscope and molecular tools toward the tremendous diversity of microbial eukaryotes, additional examples of flexible life histories will further expand our understanding of the rules of genome evolution across the eukaryotic tree of life.

## Data Availability

No new data were generated for this Perspective.
